# Estimating Demand for Industrial and Commercial Land Use Given Economic Forecasts

**DOI:** 10.1371/journal.pone.0091991

**Published:** 2014-03-19

**Authors:** Filipe Batista e Silva, Eric Koomen, Vasco Diogo, Carlo Lavalle

**Affiliations:** 1 Institute for Environment and Sustainability, Joint Research Centre, Ispra, Italy; 2 Centro de Estudos de Geografia e Ordenamento do Território, Porto, Portugal; 3 Department of Spatial Economics/SPINlab, VU University Amsterdam, Amsterdam, The Netherlands; Cinvestav-Merida, Mexico

## Abstract

Current developments in the field of land use modelling point towards greater level of spatial and thematic resolution and the possibility to model large geographical extents. Improvements are taking place as computational capabilities increase and socioeconomic and environmental data are produced with sufficient detail. Integrated approaches to land use modelling rely on the development of interfaces with specialized models from fields like economy, hydrology, and agriculture. Impact assessment of scenarios/policies at various geographical scales can particularly benefit from these advances. A comprehensive land use modelling framework includes necessarily both the estimation of the quantity and the spatial allocation of land uses within a given timeframe. In this paper, we seek to establish straightforward methods to estimate demand for industrial and commercial land uses that can be used in the context of land use modelling, in particular for applications at continental scale, where the unavailability of data is often a major constraint. We propose a set of approaches based on ‘land use intensity’ measures indicating the amount of economic output per existing areal unit of land use. A base model was designed to estimate land demand based on regional-specific land use intensities; in addition, variants accounting for sectoral differences in land use intensity were introduced. A validation was carried out for a set of European countries by estimating land use for 2006 and comparing it to observations. The models’ results were compared with estimations generated using the ‘null model’ (no land use change) and simple trend extrapolations. Results indicate that the proposed approaches clearly outperformed the ‘null model’, but did not consistently outperform the linear extrapolation. An uncertainty analysis further revealed that the models’ performances are particularly sensitive to the quality of the input land use data. In addition, unknown future trends of regional land use intensity widen considerably the uncertainty bands of the predictions.

## Introduction

The expansion of industrial and commercial land is poorly understood. Much research focuses on sector-specific dynamics and aspects such as industry location, productivity and employment [1]–[3]. The relation with land use change, however, is hardly studied. Yet this is an important aspect of the potential impact of economic development on the landscape and other environmental conditions. The development of certain economic activities requires the conversion of land from natural/semi-natural to artificial covers, often irreversibly. These dynamics are difficult to grasp: they relate to global technological and economic processes (e.g. deindustrialization of developed countries, outsourcing of production to cheap-labour countries, increased importance of information and communication technologies) as well as regional and local dynamics reflected in, for example, regional competitiveness and specialization, agglomeration economies and the performance of individual firms [4]–[9].

The understanding of the land dynamics related to industrial and commercial activities is particularly relevant for land use models that try to assess the potential future of the landscape. Land use modelling can be used to identify the drivers of land use change, and explain how these drivers and local and spatial factors interact to produce the observed landscapes. By understanding these mechanisms, past landscapes can be reconstructed given known historical records and future landscapes can be envisaged under different scenarios (assumptions on socioeconomic changes and policy alternatives). As a consequence of these capabilities, land use models have become an important element in integrated ex-ante impact assessment of policies at a wide range of spatial scales [10], [11]. Land use models, as part of a broad range of land system models, have an important role in supporting future land use policy, and may provide input for planning processes [12],[13].

In practice, land use models are used to make simulations of land use change in terms of quantity and/or location [14]. Non-spatial land use models are specialized in estimating the amount of change per land use type as country or regional aggregates, while spatially-explicit models are also able to reproduce where land use changes are likely to occur, and which local land use conversions (from one land use type to another) are expected to take place [15]. Typically, spatially explicit land use models involve the use of techniques broadly classified as cellular automata. In such models, space is represented by matrices of regular sized cells. Each cell may have a finite number of states (land uses), and these may swap over time according to a predefined set of rules regarding local and neighbour characteristics [16], [17]. On the other hand, non-spatial land use models may utilize a range of techniques, from econometric to system dynamic and agent-based approaches.

A complete land use modelling approach requires the two aspects to be integrated into a coherent framework: the estimation of the quantity and the spatial allocation of land uses for a given timeframe [18]. In most approaches, land use demand projections are computed externally and then are fed into spatial-explicit land use models for the allocation. As Verburg and Overmars [11] mentioned, this ‘top-down’ approach is necessary especially when land demand is mainly determined by “forces that are exogenous to the land allocation”. The allocation of the required land is then simulated by the geographical model considering two main dimensions: local suitability and neighbourhood interactions between the different land uses [19]. The Land Use Modelling Platform is an example of a structured platform able to integrate the two essential components of land use modelling: quantity of change and spatial allocation [20]. This platform was designed for territorial impact assessment of European policies, and can be configured project-wise, as the work by Mubareka et al. demonstrates [21].

Changes in land use quantity are often influenced by dynamics that occur at larger spatial and temporal frames and involving macro-economic and demographic changes. Therefore, the prediction of changes in land use require adequate economic context [14]. Economic models “provide a structure to represent the competition among different sectors, changes in management and technology and demand shifts due to trade or policy interventions” and are thus an important and representative input to quantify some of the drivers of land use demand [19]. As Rounsevell et al. [13] have recognized,

*innovative coupling of a range of models would allow for the consistent analysis of the land system and its interactions as a whole. The multi-model approach makes use of the strengths of existing, individual land system models and, at the same time, avoids the development of an unmanageably complex model with which to represent the whole system.*

At the continental scale, most recent land use models are now able to simultaneously simulate the allocation of more than one land use type, allowing land use competition to be represented. This means that, besides the demand for each modelled land use type, specific local suitability and the spatial interactions between land uses must be known. In models such as the CLUE-S [22] and EU-CLUE-Scanner [20], the latter aspects are addressed through a combination of empirical-statistical and rule-based approaches.

The thematic detail of models applied to small scale/large extent areas is usually restricted to just a few major groupings of land use types. Typically, artificial cover (often named ‘urban’ or ‘built-up’) is modelled as a single class, lumping together uses as distinct as residential, industrial, commercial, services, while other ‘artificial’ land uses, like transport facilities (networks, ports, airports) or green urban areas and sports and leisure facilities are kept static (i.e., not modelled) due to difficulties in mimicking the spatial dynamics underpinning such land uses through current pixel-based models. Also lacking are approaches to model industrial and commercial areas as a separate class. Having sensible methods to estimate land demands for these uses is probably the first condition to allow a split of the ‘artificial’ class into residential and industrial/commercial/services.

This paper explores and tests methodically various alternatives of estimating land demand for industrial, commercial and services land uses (from here on, we may refer to this grouping simply as ‘industrial and commercial land use’). The main focus is put into developing and testing approaches that transfer economic projections into potential land demand for a large area comprising several European countries. By studying the links between economic performance and land use dynamics, we hope also to contribute to wider and tighter integration of geography-based and economic-oriented models. Quantitative and empirical approaches will be used to explain recent expansion of industrial and commercial land, thus in line with Rounsevell et al. [13] who stated that

*empirical analysis of past and present land use change has an important role in providing insights into the socio-economic and ecological processes that shape land use transitions.* (…) For this, *quantitative data and spatial information* (…) *are necessary to detect and assess land system change, enable up-scaling of results, cross-regional comparisons and longitudinal analysis.*

In the following section, we review a selection of existing approaches to model demand for urban and/or industrial and commercial land. The subsequent sections postulate the methodology applied in this study, present a validation of the results and discuss the sensitivity to various forms of inputs and potential sources of uncertainty. The last section of this article wraps up and discusses the main findings and their implications.

## Estimation of Land Demand: Review of Existing Approaches

The estimation of future built-up area demand is usually done in the context of scenario studies but rarely the implications of the selected approaches are analysed or even discussed [23]. The appropriateness of the approaches is likely to vary according to the study area, spatial resolution and temporal scope of each application. Hoymann [23] identified three approaches to calculate demand for built-up area: trend extrapolation, regression models and density measures. In the same study, a validation exercise of the three approaches to calculate future built-up area was implemented for Germany and Czech Republic at two different spatial scales. Different models were calibrated with historical data and simulations were made for current built-up areas and then compared with observed data. One striking conclusion was that different approaches to determine future urban demand could lead to different outcomes, thus highlighting the uncertainty associated particularly with long-term projections.

Trend extrapolations apply observed growth rates of built-up area to estimate future land demand. This approach does not take into account any driving force, and simply assumes that past trends will remain constant in the future. This assumption may hold for short-term projections. However, the accuracy is expected to deteriorate with time as no causal factors are taken into account. On the contrary, regression models integrate explanatory variables to drive land use changes. Interactions between different drivers of land use change can be combined in multiple regression models. The selection of the variables to integrate the regression models are either subject to theory or automatic selection through exploratory analysis.

In the work of Reginster and Rounsevell [24] population and gross domestic product (GDP) were used as predictors of urban land use by means of a regression approach. The coefficients for the two independent variables of the regression were estimated using data from the year 2000 for Europe. Then, these coefficients were applied to different projected population and GDP in order to calculate future urban land use demand for different scenarios for the temporal range 2000–2080. However, a formal validation of the regression model was not performed. Seto et al. [25] studied the urban expansion between 1970 and 2000 at the global level by collecting and analysing results from the literature, summing a total of 326 case studies of 292 unique geographical locations distributed across the world. The annual growth rate or urban expansion was regressed against several socio-economic and locational variables. It was found that population and GDP growth rates positively influenced urban expansion, while farm subsidies, by increasing returns of agriculture land, hindered urban expansion. Results also suggested that urban growth is more sensitive to GDP growth in higher income countries than in lower income countries, and that in India and Africa, population growth is the main driver of urban expansion. The model also showed that low elevation coastal areas are more prone to higher expansion rates. Regression models to explain industrial land use have also been tested. In the work of Beckers and Schuur [26], a set of regression models establishing a relationship between employment and industrial land use in the Netherlands have been critically assessed. Their empirical findings suggest that regression models based on sectorial employment as the only predictors are insufficient to explain industrial land use, even when time lag effects and different scales of analysis are taken into account. De Vor [9], on the other hand, studied the impact of spatial factors on the location choice of industrial sites, concluding that high accessibility and economies of scale (translated in high land value and size of the working age population) are positively related to the observed supply of industrial sites in a Dutch region.

In regions where land is scarce and there is pressure to develop available land, there is a growing concern regarding the conflicts between the development of land and the protection of other assets like forested areas, farm land, landscapes and ecosystems [27]. Particularly in China, high demographic and economic growth rates of urbanizing regions have led to significant land consumption in the last two decades, thus increasing concerns about deterioration and depletion of land. Moreover, the availability of land for industrial development at low prices by local governments does not encourage an efficient use of land, leading to extensive land use and increased loss of agriculture land [28]. For these reasons, in China, as well as in other densely populated parts of the world like Japan and the Netherlands, the promotion of land use efficiency is becoming an important aspect in sustainable spatial planning [28]–[31]. In this context, measures of land use efficiency are being used by land use researchers and planners. Studies like those from Meng et al. [30] and from Huang et al. [28] have measured land use intensity/efficiency for the industrial land use in two different areas in Beijing, China. In its most simple form, land use intensity is measured as the economic output (in monetary terms) per unit of land surface. Empirical evidence collected and presented in both studies showed that land use intensity varies greatly across sectors, and that these differences may reach a factor of 40 between the least and the most land efficient sectors. In a study carried out for the whole of the Netherlands, it was also found that the average land use intensity of industrial land varies considerably across regions, and that those variations are mainly related to the sector composition of each region [31]. In line with what has been diagnosed for China, Louw et al. [31] also argued that, in the Dutch case, the supply of generous amounts of low-priced industrial land by municipalities (as a way to foster local economy) does not encourage the land use efficiency.

Intensity measures of the land use can also be used in the context of the estimation of future land demand, as proposed by Hoymann [23]. The general principle of the approach is formulated below:
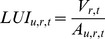
(1)where *LUI* stands for Land Use Intensity of the land use *u*, in a region *r* in the year *t*. *V* refers to a socio-economic variable related to the amount of land *A*. Consequently, the estimated land demand for land use type *u* for a year *t+n*, given *V* for *t+n* is calculated as follows:



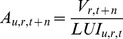
(2)To illustrate, say that, for a given region in a given time in the past, both the GDP and the industrial and commercial land use are measured in monetary and surface area terms, respectively. GDP can be obtained from official national or regional statistics, while the industrial and commercial land can be obtained either from land use maps or statistical registries. The land use intensity is obtained by dividing the GDP by the total industrial and commercial area, and expressed as units of currency per hectare of industrial and commercial use (eq. 1). Given a projected GDP for a year in the future, the amount of industrial and commercial land required to ‘support’ the expected GDP can be calculated (eq. 2). This approach assumes a stationary land use intensity over time. Yet, a dynamic land use intensity could be inferred from time-series analysis or estimated through regression techniques.

Another family of approaches to estimate future urban growth is the System Dynamics. System dynamic modelling was first introduced in the mid-1950’s by Forrester and was initially applied to solve engineering problems related to control systems in industry [32]. Soon after, the precursor of this family of models realized the potential for application to a wide range of social and economic problems, and dedicated a publication to urban dynamics [33]. This framework is suited for resolving non-linear and complex problems, allowing a representation of the behaviour of dynamic systems over time and the feedbacks between the various elements. The use of system dynamic techniques in the context of land use change modelling has become particularly popular among researchers from China. For example, Luo et al. [34] and Zheng et al. [35] have used the system dynamics approach to compute urban land demand, and then used the CLUE-S model to allocate the demands in the spatial dimension. In both studies, the demand was the result of a complex system where demography, economy and land use were interrelated. In addition, the work of Wu et al. [36] is an example of how system dynamic models can be used to make ex-ante evaluation of the impacts of scenarios of different urban land use policies. Even more recently, Lauf et al. [37] were inspired by the system dynamic principles to address the problem of modelling urban systems where growth and shrinkage occur simultaneously within the same city-region due to contradictory factors (declining population; changing of population/household structure; changing of housing preferences).

Fragkias and Geoghegan [38] developed a spatially explicit model for a county in the United States focusing on industrial and commercial land use change. The objective of this study was not so much concerned with the estimation of aggregated land demands for industrial and commercial areas, but more related to understanding the local factors affecting the discrete choices of land conversions. The underlying model is mainly econometric and land use changes are function of individual decisions to convert undeveloped into developed land parcels (residential, industrial or commercial). Two main assumptions are present in the econometric model. First, landowners seek the maximization of their earnings with respect to the net expected returns of a variety of possible conversions. Second, each land parcel has characteristics that influence both the one-time net return of the land conversion and the returns related to earnings of the land in its undeveloped state. Distance to urban centres and transportation networks/nodes, neighbourhood, environmental conditions, planning and regulations are among such characteristics. In sum, this approach could be described as purely bottom-up, whereby the land demand for industrial or commercial use is not calculated a priori, but rather the result of individual decisions over time. This exhaustive modelling approach, though, requires a wealth of detailed data which is often not available for entire countries let alone for larger regions.

In the scope of the European SENSOR project (http://www.sensor-ip.eu), an integrated approach to calculate demand for different land uses was proposed [39]. One component of the entire modelling framework was the NEMESIS econometric model, which was adapted in order to calculate endogenously demand for different land uses: agriculture, forestry, tourism, transport infrastructures, natural areas and urban. The latter is further differentiated in housing and commercial/industrial built-up. The investment in commercial and industrial buildings is computed for each given moment in time as a negative function of rental price of buildings, a positive function of production and a negative function of technical progress. The net investment in buildings for a given time *t* corresponds to the building stock, in Euros, in time *t* minus the building stock in time *t-1* times a parameter reflecting the rate of decay of buildings. The net investment is determined for over 30 economic sectors represented in the model, and then summed to obtain the total net investment in commercial and industrial buildings. A ‘technical coefficient’ transforms the total net investment, expressed in monetary terms, in actual land requirements. A similar approach is used to obtain demand for new residential land. However, the investment in housing buildings is, instead, a positive function of real disposable income, and negative functions of the real interest rates and building prices. Calibrated with data from 2000, the model is able to make estimations on demand for housing and commercial and industrial areas by first determining the net investment in buildings for any given year. The land demands are computed at the country level and then passed on to the CLUE-S model for spatial allocation at the resolution of 1 km^2^ [39]. An important characteristic of this approach is the linkage between the economic dynamics and its consequences in terms of potential land uptake, as well as the consideration of feedbacks in the process. As a result, the land claims of the different sectors “[are] price elastic to the extent that they will respond negatively to any increase in building price” [40].

In a nutshell, and despite all the progress made so far in the field of land use change, specific focus on the industrial and commercial areas has been limited so far. Models addressing dynamics of industrial and commercial land are usually applied at local scales and/or require data inputs inaccessible or even non-existent at continental scales. In addition, formal validations of the various approaches are lacking. Yet, nowadays, policy support at supra-national level demands more integrated assessments together with more spatial and thematic detail.

## Methods to Estimate Demand for Industrial and Commercial Land

The objective of this study is to develop and validate approaches to estimate demand for industrial and commercial land. The approaches should be relatively straightforward so that they can be easily replicable and applicable to large spatial regions (e.g. countries and continents) in the context of land use modelling. We propose to explore in particular those approaches based on land use intensity measures. The main reason for this choice relies on the fact that, as reviewed in the previous section, these measures are not especially intensive to calculate, requiring only a few aggregate variables, characteristics which become relevant when working at very large spatial extents. Still, intensity measures are informative and conceptually easy to interpret. Moreover, they link to sector-specific processes of economic development that are expected to be relevant for the land uptake of industrial and commercial use. Using intensities allows linking land use simulations to regional economic projections.

Below in this section, several variants of the land use intensity approach are formally introduced. By definition, intensity measures integrate one driver of land use change at a time. In this study, sector gross value added (GVA) is used as a proxy for sector economic output. In addition to the land use intensity approaches, trend extrapolations are also tested. Trend extrapolations can be seen as the simplest way to make estimations because they do not specifically address drivers of land use change, but rather apply observed growth trends to describe possible future conditions. The main reason to consider trend extrapolations in this study is to create an adequate term of comparison for the estimations based on intensity measures.

Once introduced, the models will be applied to estimate the demand for industrial and commercial land for a set of countries in Europe.

### Models Description

#### Trend extrapolation (models 1 & 2)

**Figure 1:**
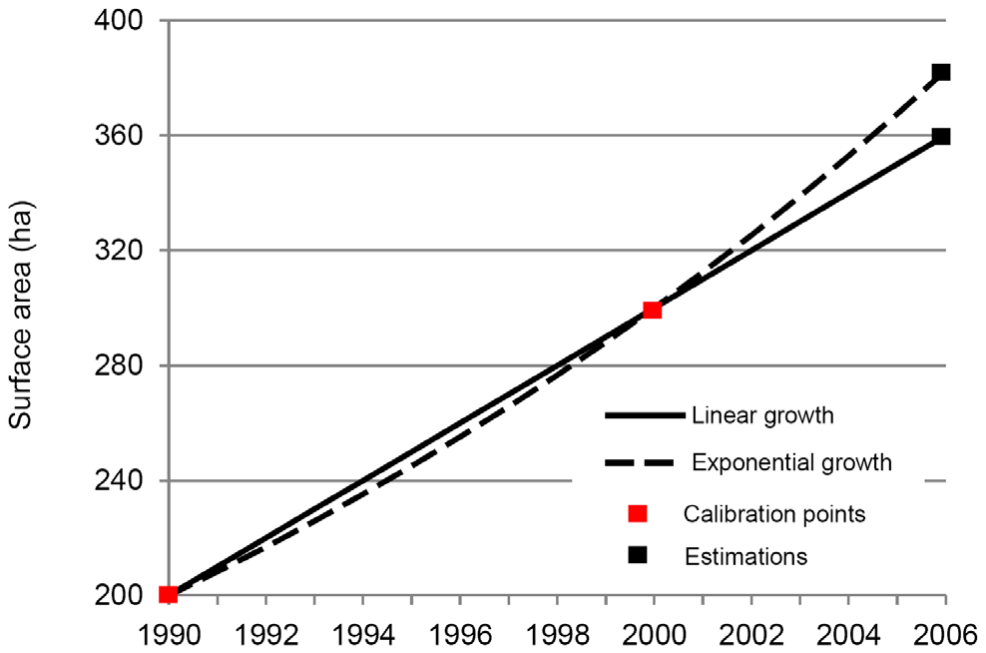
Extrapolation models for hypothetical region with 200 and 300 hectares of industrial and commercial land in 1990 and 2000.



Two trend extrapolation methods are considered: a linear extrapolation (model 1) and an exponential extrapolation (model 2). The linear extrapolation is formulated in equation 3:
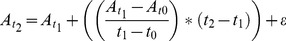
(3)where *A* refers to the industrial and commercial area, *t_0_* and *t_1_* correspond to the starting and ending years of the calibration, respectively, and ε is the error term. In this method, the average yearly absolute growth of the calibration period (*t_0_* to *t_1_*) is multiplied by the total forecasting years (*t_2_–t_1_*) to obtain the estimate for desired year *t_2_*. In the exponential extrapolation, the average yearly growth rate *G* observed between *t_0_* and *t_1_* is firstly obtained through equation 4, and it is then applied to estimate the industrial and commercial land in *t_2_* (equation 5). The graph in Figure 1 shows the application of both models to a hypothetical region with 200 ha of industrial and commercial land in 1990, and 300 ha in 2000.

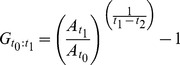
(4)

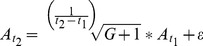
(5)


#### Region specific land use intensity (models 3 & 4)

The economic product and surface area of commercial and industrial uses are consistently highly correlated at the regional level. If we take the sum of the regional gross value added of the industrial, commercial and service sectors and relate it to the respective surface area as reported by the CORINE Land Cover (CLC) datasets, correlation coefficients ranging between 0.74 and 0.76 can be found for the years 1990, 2000 and 2006, in Europe. This suggests that, in general, the higher the economic product of a region, the more physical infrastructure is required to support the economic activity.

Models 3 and 4 are characterized by using economic output or product *P* of regions as the driver of development of industrial and commercial areas. In both models, a land use intensity approach is used to relate the economic product with the respective area of industrial and commercial units. In model 3, the land use intensity LUI is computed for the year *t_1_* and measured as economic output per hectare of industrial and commercial land (eq. 6). Then, assuming a stable land use intensity in time, and knowing the product *P* for *t_2_*, the total industrial and commercial land is predicted (eq. 7).
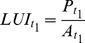
(6)




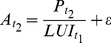
(7)


This model takes the whole regional product and the whole existing area of industry and commerce per region in *t_1_* to compute the land use intensity. However, the total amount of industrial land is strongly related to historic developments and only partly dependent on current economic performance. In fact, as existing industrial and commercial land is likely to remain (with or without actual economic activity), this inertia is not captured by a single and static snapshot of the land use intensity. So we should perhaps focus especially on changes in economic development and related changes in the amount of land needed. This implies that the land use intensity of new developments is important in order to capture shifts in the production structure. Model 4 builds upon this idea. It measures land use intensity only of the industrial and commercial land developed during the calibration period *t_0_* and *t_1_* (eq. 8). The ‘land use intensity of the recently developed land’ is then used to estimate the extra land related to the growth of the product in the subsequent period (*t_1_:t_2_*) (eq. 8). Contrary to the model 3, this approach ignores the land use intensity of the industrial and commercial land developed prior to *t_0_*.
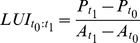
(8)




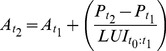
(9)


We call these approaches ‘region specific’ because the intensity measures described above can be computed separately for any set of regions composing the whole of any area of interest. Consequently, regional differences in land use intensity (which are underpinned by differences in productivity and production structure) are captured.

#### Region and sector specific land use intensity (models 5 & 6)

Industrial and commercial land is a rather broad and heterogeneous land use class. For example, in the CORINE Land Cover nomenclature, the homonymous land use class includes factories of all different kinds of industries, facilities for energy production and telecommunication networks, facilities related to defence and security, shopping malls and exposition sites, and a wide range of facilities related to public or private services likes schools, university and research campuses and hospitals [41]. Trying to model such a heterogeneous class as a whole poses obvious limitations. Most obvious of all, land use intensities vary considerably among industries [28], [30], [31], let alone the differences between the various economic sectors.

To address this limitation, one could think of making the land use intensity measures both region and sector specific. In this case, the economic product of a given sector *s* would be related to the land area *A* known to be used by sector *s* in year *t_1_* (eq. 10). At this point, it would be possible to estimate the aggregated industrial and commercial land for a given *t_2_* (eq. 11). Conceptually, this formulation is more robust than models 3 and 4 because it allows the integration of land use intensities specific to *n* number of sectors (model 5). In addition, a factor *ω* could be used to transform the land use intensities when calculating *A* in *t_2_*, as a function of the observed changes in LUI between *t_0_* and *t_1_*, that is *ω_s_* = f(ΔLUI_s,t0,t1_). In this study, we let *ω* = 1 for all sectors.
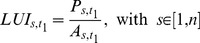
(10)




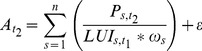
(11)


This model can be also combined with the concept of ‘land use intensity of the recently developed land’, as introduced in model 4. This is done by applying equations 12 and 13 (model 6):
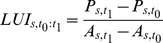
(12)




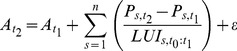
(13)


## Case Study: Estimation of Industrial and Commercial Land Use in Europe

### Overview of the Case Study Set-Up

The case study consists of estimating the amount of industrial and commercial land use in Europe and comparing the estimates against reference land use data. Estimates will be produced by each of the six models described in the previous section and listed in table 1, for a set of Central and Western European countries. Countries from Scandinavia, the Balkans Eastern Europe and the UK were not included in the analysis due to incomplete land use and/or economic data time-series. The spatial unit of analysis used was the NUTS2 regions. The NUTS, or Nomenclature of Territorial Units for Statistics, is the European Union’s official regional subdivision of member states, comprising three hierarchical levels (NUTS1, NUTS2 and NUTS3).

**Table 1:** Main model characteristics.

Modelnr.	Family of approach	Driver of landuse change	Calibrationyears	Recent land useintensity	Sector specificLUI	Equations
**M1**	Trend extrapolation (linear)	None	1990, 2000	Not applicable	Not applicable	3
**M2**	Trend extrapolation (exponential)	None	1990, 2000	Not applicable	Not applicable	4 & 5
**M3**	Land use intensity measures	Gross Value Added	2000	No	No	6 & 7
**M4**	Land use intensity measures	Gross Value Added	1990, 2000	Yes	No	8 & 9
**M5**	Land use intensity measures	Gross Value Added	2000	No	Yes	10 & 11
**M6**	Land use intensity measures	Gross Value Added	1990, 2000	Yes	Yes	12 & 13

In order to measure the predictive power of land change models, Pontius et al. [42] and Pontius and Malanson [43] recommended that the calibration and validation should be separated processes, and that the modelling results should be compared to a ‘null model’. The null model predicts pure persistence, i.e., no change during the modelling time span. In line with these recommendations, each of our models is calibrated using historical data for two points in time, *t_0_* and *t_1_*, and is then used to estimate industrial and commercial land for a third point in time, *t_2_*. Finally, all models (including the null model), are compared in terms of their ability to predict the actual total industrial and commercial land for *t_2_* as reported in a reference data source.

The following subsection will focus on the data used to feed the models and generate the estimates. Finally, the indicators used to measure the model performances are presented, and the results are reported and commented.

### Data

#### Economic data

Gross value added per sector of activity was collected from Eurostat’s online database (http://epp.eurostat.ec.europa.eu) at regional level (NUTS3). A time-series comprising the period 1985 to 2009 was compiled. All values were initially collected in current prices in Euros. The existing gaps were filled by using United Nations (UN) data (http://data.un.org), which were available in current US Dollars at country level only. For the missing years in the Eurostat database, annual growth rates were derived from the UN data, and then applied to generate country level data in Euros. Finally, the country values were regionally disaggregated using the regional shares of the closest available year in Eurostat. For the specific purpose of the case study, two additional procedures were applied. The values in current prices in Euros were transformed to constant prices as of 2005. The economic output expressed in constant prices is more suitable for time-series analysis because the effect of inflation is removed, thus reflecting the actual economic growth. Finally, the NUTS3 values were aggregated to NUTS2 to match with the spatial unit of analysis used in this study. GVA from three main categories of economic activity was used: industry; commerce and private services; and public services and administration.

#### Land use data

The source for ‘industrial and commercial land’ is the CORINE Land Cover (CLC). The three available editions (1990, 2000, 2006) were used. In the context of this case study, we considered 1990 as *t_0_*, 2000 as *t_1_* and 2006 as *t_2_*. The maps produced in the context of the CLC project are the only datasets providing a time-series of land use change that are consistent across European countries [44], [45] because common nomenclature and standard methodology guidelines were used in its elaboration [41], [46]. However, one major disadvantage of CORINE Land Cover is related to the thematic detail of its nomenclature. As mentioned earlier, the CLC class ‘industrial and commercial’ land use class aggregates a broad range of land use sub-categories that are not distinguishable by any further breakdown. As a result, CLC alone does not provide the minimum necessary sectorial detail to implement models 5 and 6.

To address this limitation, we focused on two countries in more detail. These countries were selected based on their different economic structure and the availability of detailed land use datasets and comprise of Spain (Sistema de Información de Ocupacióm del Suelo en España, http://www.siose.es/siose) and the Netherlands (Statistics Netherlands, http://www.cbs.nl). To calculate sector specific land use intensities, we first had to correspond the broad economic sectors with land use classes found in the Spanish and Dutch land use maps (see table 2). This correspondence allowed us to compute the land use intensities for the three broad economic sectors *s* for the Spanish and Dutch NUTS2 regions, using equation 10 where the economic product *P* was represented by the GVA. The land use intensities were computed for *t* = 2005 in the case of Spain and for *t* = 2006 in the case of The Netherlands. These years were chosen in order to match the reference dates of the national land use data sources.

**Table 2:** Correspondences between broad economic sectors and land use nomenclatures (SIOSE and CBS).

Broad sector label	Land use classes (SIOSE, Spain)	Land use classes (CBS, Netherlands)
**Industry**	Industry (821, 822, 823); mining and quarrying (833); energy (891, 892,893, 894, 895, 896); water supply (911, 913)	Business estates (24); mining area (33)
**Commerce and private** **services**	Commerce and offices (841); hotels (842); recreation parks (843); camping (844)	Retail and catering (21)
**Public services and** **administration**	Public administration (851); health (852); education (854); penitentiary (855)	Public facilities (22); socio-cultural facilities (23)

Between brackets are the respective class codes of both Spanish and Dutch land use maps.

We found that LUI_commerce_>LUI_services_>LUI_industry_ for all regions, i.e., the highest economic output (GVA) per unit of land occurs in the commerce sector, followed by the services sector and by the industry sector. This relationship can also be interpreted as the area necessary to produce the same monetary unit, which is highest in the ‘industry’ sector and lowest in the ‘commerce’ sector. To find whether these patterns were consistent across regions, the coefficient of variation CV was computed for each sector *s* and country *c*, with σ_LUI_ and μ_LUI_ being the standard deviation and the average of the land use intensity, respectively (see eq. 14).
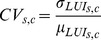
(14)


Table 3 presents the average land use intensities per sector and per country and the respective coefficient of variation. The results show a relatively small variance of the land use intensities of each sector within each country (CV <1 for all sectors in both countries). In addition, considering Spain and Netherlands altogether, we could infer that the commerce and service sectors are, on average, 27.6 and 6.7 times more land use intense than the industry sector (see values between brackets in table 3). These values can be interpreted as ‘land use weights’. The higher the ‘weight’ the higher the land use intensity and, therefore, the less land required to produce one monetary unit of GVA.

**Table 3:** Land use intensities and coefficient of variation (CV) per sector of main economic activity and per country.

	Industry		Commerce and private services		Public services and administration	
Country	LUI (M€/Yr*ha)	CV LUI	LUI (M€/Yr*ha)	CV LUI	LUI (M€/Yr*ha)	CV LUI
**Spain**	0.53 (1.0)	0.58	14.47 (27.1)	0.46	4.83 (9.0)	0.28
**Netherlands**	1.16 (1.0)	0.43	33.92 (29.1)	0.42	4.14 (3.6)	0.33
**Spain+Netherlands**	**0.67 (1**.0**)**	**0.70**	**18.60 (27.6)**	**0.69**	**4.53 (6.7)**	**0.32**

**Note**: Values between brackets correspond to each sector’s land use intensity in respect to the industry’s land use intensity (LUI_s_/LUI_industry_).

The above empirical findings can be used to make an estimation of the sector composition of the ‘industrial and commercial’ class of CLC, i.e., how much of the whole land classified as ‘industrial and commercial’ in CLC refers to the generic sectors ‘industry’, ‘commerce’ and ‘services’ individually. At first, we weighted the sector product (GVA) by the sector land use weights *w_s_* (eq. 15). The area of each sector is then calculated by multiplying the whole ‘industrial and commercial’ area (as reported in the CLC) by the estimated share (eq. 16). By definition, A_t_ = ∑_s_ A_s,t_. This procedure was applied to ‘disaggregate’ the CLC class ‘industrial and commercial’ and thus obtain estimates of its sectorial composition for the calibration years *t_0_* and *t_1_* for all regions covered in this study.
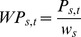
(15)




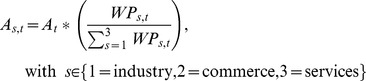
(16)


### Validation Results

**Figure 2:**
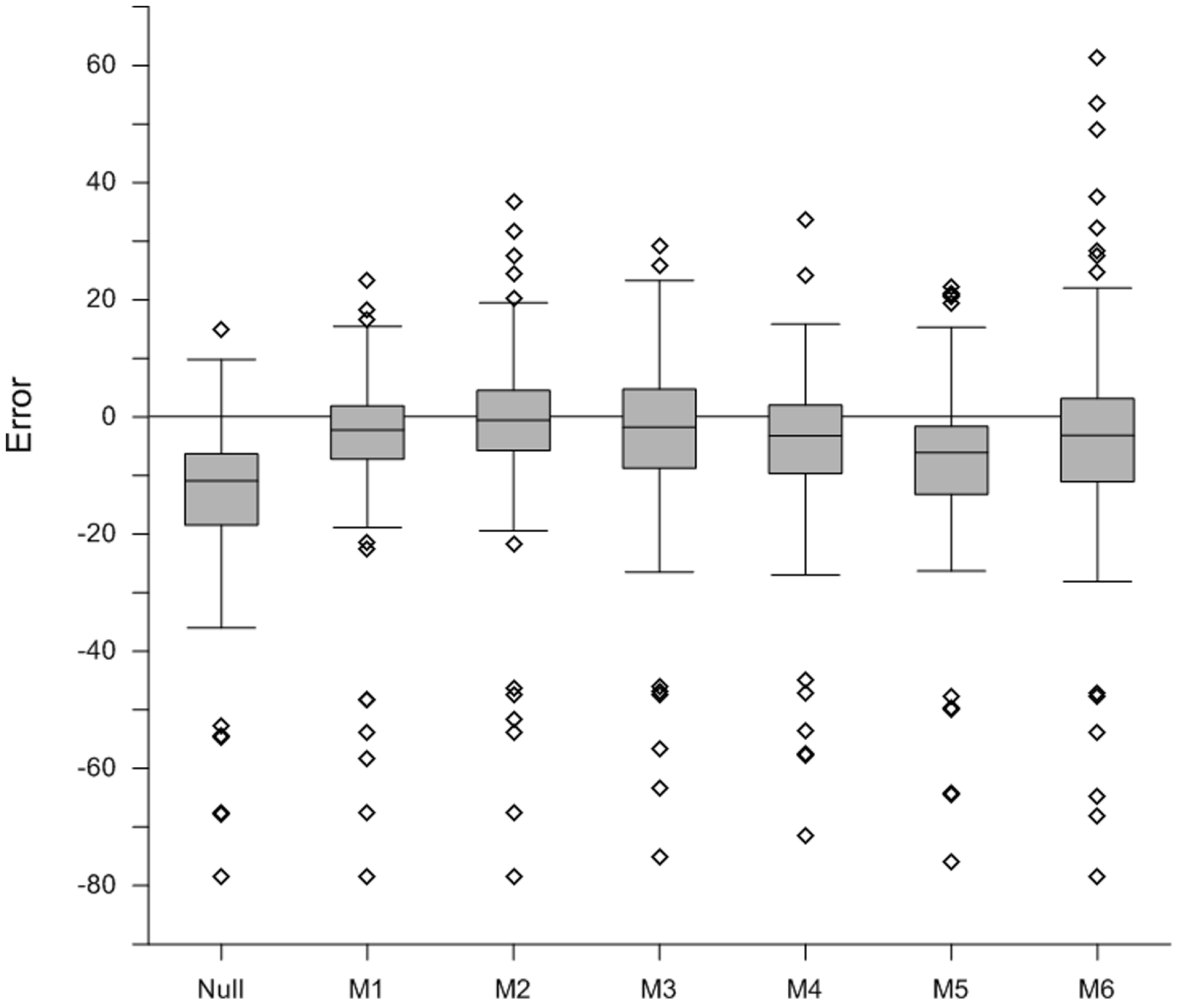
Distribution of the errors for each model (%).

All models were calibrated with data up to the year 2000 and were then applied to estimate the industrial and commercial land in 2006. The validation is done by comparing each model’s estimates with the actual amount of industrial and commercial land as reported by the CLC 2006, which is the nearest available to ground truth for the whole study area. The indicators used to measure the performance of the models are summarized in table 4 and the results can be consulted in tables 5 and 6 and in Figure 2.

**Table 4:** Validation indicators computed for each model.

Indicatorname	Short description	Formula
Relative difference (RD)	Relative difference between the estimated and the observed industrial and commercialarea for the whole study area. It shows the magnitude of the aggregated deviationas well as the sign of the deviation. Negative and positive values mean underand overestimation, respectively. Expressed as percentage.	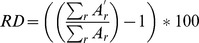
Average Absolute Error (AAE)	Average of all absolute regional deviations. It is always positive.Expressed in hectares.	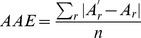
Total Absolute Error (TAE)	Sum of all absolute regional deviations. It is expressed as percentage of the totalknown industrial and commercial land in 2006. It is always positive.	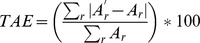

**Note**: A – Known industrial and commercial area in 2006 (as reported in CLC2006); A’ – Estimated industrial and commercial area for 2006; r – NUTS2 region; n – total number of NUTS2 regions.

**Table 5:** Validation results.

Model nr.	RD (%)	AAE (ha)	TAE (%)
**Null**	−11.68	1033	11.75
**M1**	−2.70	501	5.70
**M2**	−0.56	563	6.40
**M3**	−2.03	571	6.49
**M4**	−3.36	631	7.18
**M5**	−7.06	700	7.97
**M6**	−2.55	854	9.72

**Table 6:** TAE per country (%).

	Models						
Country	Null	M1	M2	M3	M4	M5	M6
Austria	35.5	31.9	31.4	27.7	30.4	29.5	31.4
Belgium	5.7	6.8	9.2	8.0	6.1	2.8	2.0
Germany	8.9	4.5	5.0	5.1	4.9	6.9	11.9
Denmark	9.6	3.6	3.7	2.3	3.7	6.2	5.7
Spain	17.7	7.1	7.8	6.3	8.7	10.9	13.0
France	8.4	3.3	3.3	4.2	4.0	3.7	4.5
Ireland	37.1	23.3	17.1	15.9	23.3	31.8	34.6
Italy	13.2	7.2	7.8	8.0	11.1	10.4	11.8
Luxembourg	23.7	18.0	17.3	3.0	17.9	15.3	17.4
Malta	4.3	11.3	12.0	19.6	7.9	13.7	8.3
Netherlands	17.2	5.4	7.3	9.4	7.2	11.5	11.0
Portugal	15.0	5.1	12.4	8.5	10.4	10.6	11.8
Slovenia	0.9	0.3	0.3	25.9	0.1	21.5	0.5

Results reported in table 5 show that all models performed better than the null model which tell us that modelling demand for industrial and commercial areas appears to be a worthwhile exercise. However, all models have more or less underestimated the total amount of industrial and commercial land in 2006. While models 2 and 3 have best approximated the absolute expansion of land use for the whole study area, it can also be concluded that no model sufficiently reproduced the actual observed growth for the period 2000–2006. Nonetheless, most of the underestimations fall in a relatively narrow range, from −0.56% to −3.36%, and −7.06% in the worst case.

Overall, model 1 seems to be the best performer, as it scored best for AAE and TAE, and also showing one of the lowest relative differences. In addition, this model also shows the narrowest distribution of errors (see Figure 2). This indicates that the models that incorporated the economic output as driver for land use change were not able to perform better than trend extrapolations. Among the models that use GVA as driver of land use change, model 3 stands out, as its predictions are overall closer to the known land use in 2006 than predictions from the others. Models 4 and 6, which used the land use intensity of the land developed in the period 1990–2000 to estimate the land developed in 2006 performed worse than models 3 and 5, respectively, the latter using the overall land use intensity of all existing industrial and commercial land use. Finally, the models which integrated both regional and sectorial specific land use intensities (models 5 and 6) did not outperform the models which relied only on an overall land use intensity per region.

Performances vary significantly country wise (table 6). Countries like Belgium, Denmark, France, Germany and Spain show fairly low errors for most modelling approaches, whereas the estimations for Austria, Ireland and Luxembourg were overall much worse, with all models severely underestimating the observed land use expansion. The negative outlier points identified in the box-whisker plot (Figure 2) correspond to NUTS2 regions of the latter countries. Even though the linear extrapolation (model 1) showed the lowest overall estimation errors, model 3 performed best for Austria, Denmark, Spain, Ireland and Luxembourg. In addition, models 4 and 5 obtained second best estimations for a number of countries.

#### Sensitivity to land use data

As all models strongly depend on land use data, we can expect the final results to be very sensitive to the accuracy of such input. Biases and inaccuracies in the reporting of observed land use propagate to the land use intensity measures which thus influence the final land use demand estimation. Regional industrial and commercial land use areas were derived from CLC data, which covers all Europe with a time-series comprising the years 1990, 2000, and 2006. Despite the common nomenclature and mapping methods, temporal and spatial inconsistencies have been reported [45]. Moreover, the large minimum mapping unit (MMU) of 25 hectares may create mapping artefacts. For example, land use patches smaller than the MMU in *t_0_* are ‘hidden’ within the dominant surrounding land use patch of another land use class. If the former patch expands to an area above the MMU in *t_1_*, the patch is then mapped, thus giving the impression of an overestimated land use expansion between *t_0_* and *t_1_*.

To test whether the results obtained in the validation were influence by CLC data issues, we have applied the same six models to the Dutch regions using finer land use time-series data from a national source (Statistics Netherlands, http://www.cbs.nl). The economic data was kept the same, as well as specifications for all six models. A comparison of modelling errors for the Netherlands using CLC and CBS land use data is reported in table 7. Two major conclusions can be drawn from the figures presented. First, there is a clear improvement of performances of all models when using finer land use data from a national source. Moreover, the proportion of performance improvement to the null model is higher when using finer land use data. Second, the consistent underestimation of land use demand when using CLC data is not observed when using finer data. This leads us to infer that issues directly related to the land use data partly explain the consistent model underestimation for the European case study. More specifically, these results may indicate that CLC underestimated the amount of industrial and commercial land use in 1990 and 2000 in relation to 2006, thus contributing to an overestimation of the land use intensities in 1990 and 2000.

**Table 7:** Validation results for Netherlands using different land use sources (CLC and CBS).

Model nr.	RD (%)		AAE (ha)		TAE (%)	
	CLC data	CBS data	CLC data	CBS data	CLC data	CBS data
**Null**	−17.16%	−6.93	1163	734	17.16	6.93
**M1**	−2.58%	1.44	364	214	5.38	2.02
**M2**	3.87%	2.24	495	282	7.31	2.66
**M3**	−9.13%	1.42	637	261	9.41	2.47
**M4**	−5.87%	−3.75	490	397	7.24	3.75
**M5**	−9.12%	−1.13	657	369	9.70	3.48
**M6**	−0.24%	−6.14	742	751	10.95	7.09

In addition to these aspects, we must acknowledge temporal nonstationarities that might be present in the real world but which are not captured by any of the models. For example, in certain regions, spatial planning policies driven by expected economic growth may have led to oversupply of business estates that remain empty, thus decreasing the land use intensity in 2006. Other economic dynamics, such as changes in economic structure (e.g. shifts from labour intensive to capital intensive industries), can lead to appreciable changes in the land use intensity of regions over time. The uncertainties related to the future trends in regional land use intensities will be addressed in the following chapter.

## Analysis of Land Use Uncertainty

It has been noted that “when model parameters are fit by calibration to historical data, additional uncertainty is introduced due to the inherent temporal nonstationarity of processes” [47].

In this section we explore the main sources of uncertainty related to our modelling approach and their implications in terms of predicted land use demand. As explained in chapter 3, we proposed a deterministic method to estimate future demand for industrial and commercial land uses. The method relies on a single parameter, the land use intensity, which is calculated based on regional data on land use and economy. The land use intensity is expressed in terms of gross value added per hectare of industrial and commercial land in a given year in each region. This parameter is then assumed to remain constant in time and it can be used to predict future demand for industrial and commercial land given regional economic projections. The total uncertainty of the resulting land use demand predictions includes both the uncertainty of the land use intensity parameter and the uncertainty of the economic projections.

In this paper we focus only on the uncertainty of the land use intensity parameter. The uncertainty of economic projections is beyond the scope of this paper, as it is a field of research in its own. Moreover, the primary concern of this study is to design and test methods to translate given economic projections into future demand for industrial and commercial land use. The economic projections are herein dealt as exogenous assumptions, whose uncertainties shall be estimated in the appropriate framework of the economic modelling. Under this premise, the uncertainty of the estimates of future land use demand boils down to the land use intensity parameter. The uncertainty related to this parameter exists in two forms: first, the uncertainty of the measurement itself for a given moment in the past; second, the uncertainty regarding its future evolution. The sections below will focus on each of these two aspects of uncertainty.

### Accounting for Land Use Mapping Errors

The land use intensity of a region is determined by dividing the regional GVA by the regional land use acreage. The latter is normally described in the form of spatially explicit land use maps, while the former is typically reported by governmental agencies according to international conventions. In Europe, Eurostat – the official statistical body of the European Commission – ensures standardization and discloses GVA data for all European countries and regions. Figures about the state of the economy can be subject to various distortions, such as measurement errors, intentional biases from reporting entities, exclusion of the parallel (not officially registered) economy. The uncertainty of the national accounts figures is, however, not communicated and thus cannot be included in this analysis. Therefore, we focus our uncertainty analysis on the errors associated with the land use maps we apply and that relate to aspects such as classification errors, and minimum mapping unit. The latter issue is particularly critical when using CLC data, as demonstrated previously.

Notwithstanding the importance of CLC as the sole European-wide land use/cover map, there has been limited reporting on its quality. Only the 2000 version of this dataset was subject to an extensive and systematic validation [44]. The thematic accuracy of CLC 2000 was assessed by comparing its classification with a classification derived from a field survey carried in the year 2000, the land use/cover area frame survey, better known as LUCAS. However, the validation yielded statistically inconclusive results for a number of land use classes for which the sample size was particularly small. This was the case of the land use class ‘industrial and commercial units’, for which only 34 points were controlled in all Europe.

To obtain an idea about the mapping errors in CLC we, therefore, rely on a statistically sound validation of CLC 2006 that was performed for one specific country [48]. In this validation effort for Portugal, a stratified random sampling scheme was adopted, with 100 sample points randomly selected for each land use class, in order to guarantee “a representative and meaningful basis for accuracy assessment” [48]. For each sample point, the mapped land use class was compared with visual observations of land use, enabling the construction of a ‘contingency table’ (or ‘confusion matrix’) which allows map accuracy indicators to be calculated [49]. The contingency table for the Portuguese CLC 2006 is reported in the work of Caetano et al. [48].

The binomial distribution is often applied to discrete land use classifications because each land use class can either occur or be absent at each location. When the sample size is large enough, it can be assumed that the proportion of errors of a land use class with the other land use classes is normally distributed. This allows the confidence intervals of land use accuracy assessments to be estimated through the use of a normal approximation of the binomial distribution. Given the large sample size, this approach is recommended by Cochran [50], and was adopted, for instance, by Card [49], EEA [44], and Carrão et al. [51]. By applying the detailed formulas presented by Carrão et al. [51] to the validation figures reported in the contingency table from Caetano et al. [48], we were able to estimate the true total area of the industrial and commercial land use for each Portuguese region, and the respective variances. The unbiased estimate of the true total land use and the respective estimated variance for each region depends upon the confusion between land uses presented in the contingency table and the abundance in each region of land use classes with which the industrial and commercial classes are confused with. After estimating the variance, confidence intervals can be drawn for each region around the estimated true total area of the industrial and commercial land use. Because a normal approximation of the binomial distribution was adopted, the confidence interval associated with each estimated parameter (i.e. true industrial and commercial land use in each region) is symmetric.

**Figure 3:**
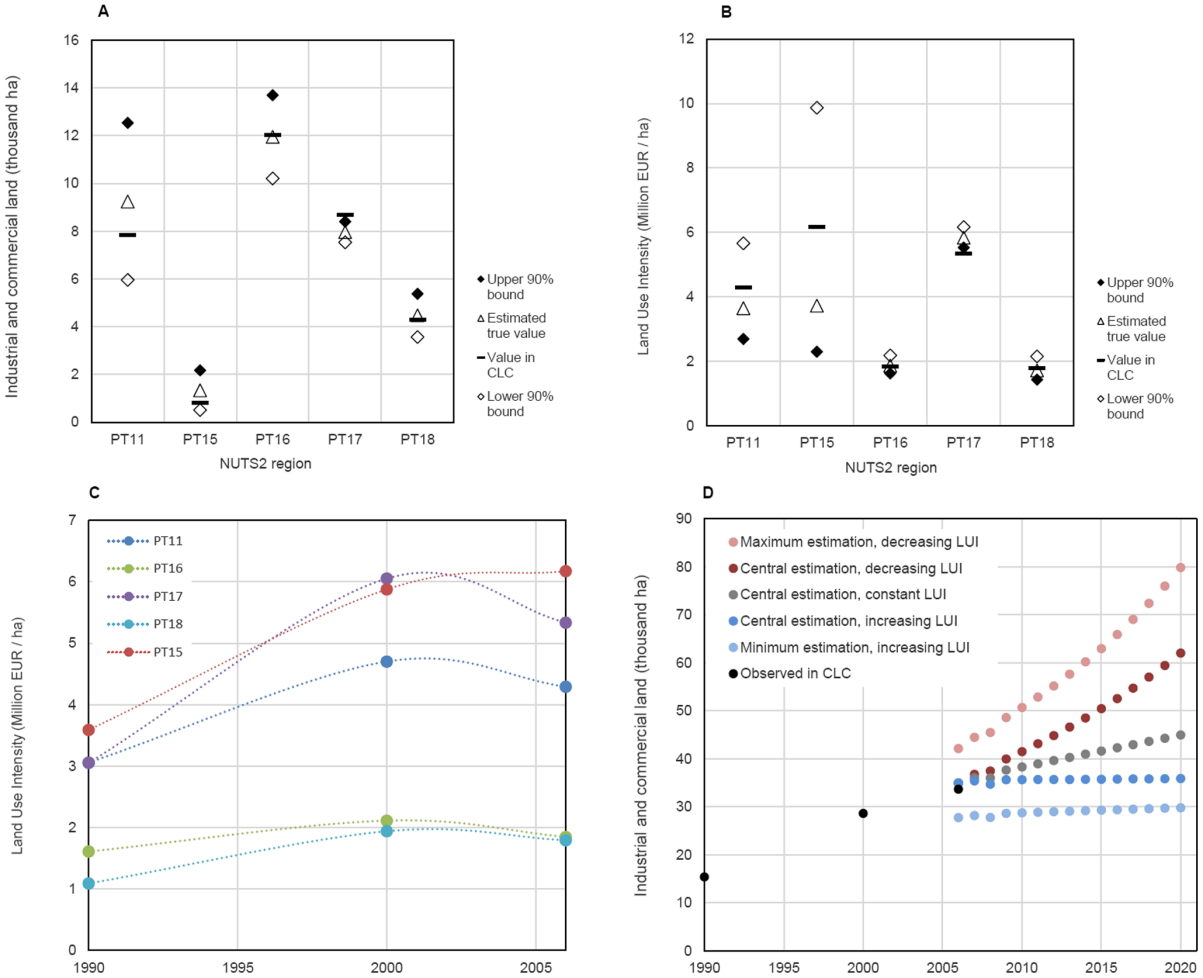
A: Industrial and commercial land use in 2006 per region, with 90% confidence interval. B: Land use intensity in 2006 per region, with 90% confidence interval. C: Land use intensities 1990–2006 per region. D: Scenarios of future demand for industrial and commercial land use (sum of all Portuguese regions).

The results for Portugal are depicted in Figure 3A. For each NUTS2 region, it shows the amount of industrial and commercial sites mapped in CLC, the estimates of the true area of industrial and commercial land use, and its 90% confidence interval. If we take as example the region PT16, we find that the amount of industrial and commercial sites reported in CLC approximates the estimated true value, and that it is 90% likely to find the true value between 10.2 and 13.7 thousand hectares (based on the validation sample).

The uncertainty in the accounting of the industrial and commercial land use propagates to the land use intensity parameter. As such – and assuming that the GVA figures are correct, as discussed earlier –, the distribution of the land use intensities for the Portuguese regions is depicted in Figure 3B. The regions PT11 and PT15 are particularly sensitive to the uncertainties regarding the true amount of industrial and commercial land use, while PT16, PT17 and PT18 are considerably less sensitive.

Summing up, in this section we looked at the uncertainty of the land use intensity in an indirect way: we first assessed the uncertainty of the industrial and commercial acreage reported in the land use map (figure 3A), and then looked at the impact of such uncertainty in the actual measurement of the land use intensity (figure 3B). Higher relative uncertainties of industrial and commercial acreage cause higher uncertainties on the true land use intensity. In the next section, we will combine the uncertainty of the land use intensity parameter (as just discussed) with the uncertainty of its future evolution.

### Accounting for Nonstationarity in Land Use Intensity

To account for nonstationarity in land use intensity we analysed temporal changes in observed intensity figures for the Portuguese regions. We focus on this country as it allows us to assess the impact of nonstationarity in relation to the mapping error addressed in the previous section. Figure 3C shows the measured land use intensity of the Portuguese regions for the years 1990, 2000 and 2006, using data from Eurostat and the CLC time-series. We can observe an increase in intensity from 1990 to 2000, followed by a slight decrease between 2000 and 2006.

In studies by Chen et al. [29], Meng et al. [30], and Louw et al. [31], increases in land use intensity over time were observed for different study areas. Regional differences in land use intensity have been attributed to agglomeration economies, differences in economic structure and production characteristics. In addition, Louw et al. [31] argued that policy-related factors influence land use intensity, in particular by interfering with the supply of industrial land. The existing research, however, does not provide a solid framework to anticipate future changes in land use intensity for the European regions. To account for this uncertainty, we constructed two extreme trends for the future evolution of the land use intensity. One trend assumes that the land use intensity will continue to increase as observed in the period 1990–2006 for each respective region. The other assumes that the land use intensity will decrease as observed in the period 2000–2006. These two variants for the unknown evolution of the land use intensity are seen as bracketing the likely future values, thus providing a worst-case scenario for the uncertainty range of future land use intensity.

To illustrate the potential variance in estimates of future land use demand, we apply our demand model 3 to a scenario in which the economic output of regions is assumed to grow linearly until 2020, as observed in the period 1995–2008. Based on these premises, we constructed five possible trends of future industrial and commercial land use demand:

- Trend 1 (central estimation, constant land use intensity). The estimated true land use intensity for 2006 and for each region remains constant in time.
- Trend 2 (central estimation, increasing land use intensity). The estimated true land use intensity for 2006 increases in the same pace as observed in the period 1990–2006 in each region.
- Trend 3 (central estimation, decreasing land use intensity). The estimated true land use intensity for 2006 decreases in the same pace as observed in the period 2000–2006 in each region.
- Trend 4 (maximum estimation, decreasing land use intensity). The upper endpoint of the 90% confidence interval for the land use intensity for 2006 decreases in the same pace as observed in the period 2000–2006 in each region.
- Trend 5 (minimum estimation, increasing land use intensity). The lower endpoint of the 90% confidence interval for the land use intensity for 2006 increases in the same pace as observed in the period 1990–2006 in each region.

Figure 3D shows the resulting aggregated demand for industrial and commercial land use for Portugal. Trend 1 is a typical central and deterministic trajectory, which does not incorporate the uncertainties related to the land use mapping and assumes a stationary land use intensity over time. Trends 2 and 3 incorporate the uncertainty related to the future evolution of the land use intensity, thus providing lower and upper bounds for the future land use demand assuming we are certain about the measured land use in 2006. In 2020, the estimated value in trend 3 is circa 1.7 times higher than the one estimated in trend 2. Finally, trends 4 and 5 incorporate the uncertainty related to the future evolution of the land use intensity plus the uncertainty regarding the true acreage of industrial and commercial in 2006. In 2020, the estimated value in trend 4 is circa 2.7 times higher than the one estimated in trend 5.

These trends were constructed in order to translate the likely maximum variance of future demand for industrial and commercial land use, avoiding any underestimation of uncertainties. These results indicate that the uncertainty band for the projected land use is rather large, which is not surprising given the coarse resolution of the CLC and the unforeseen trajectories of future land use intensity.

## Discussion and Conclusions

Estimating demand for future industrial and commercial land use is a challenging exercise. Very few attempts are known in the literature, and, when attempted, demands are estimated for small study areas, with detailed input data on land use and economy. In this study we aimed at developing and testing straightforward methods to estimate demand for new industrial and commercial land at continental scale with sub-national detail. The main difficulties concerned the input land use data, the CORINE Land Cover, which provides low spatial detail (minimum mapping unit of 25 hectares) and low thematic detail (the industrial, commercial and services land uses are all lumped together in one single land use class). The CLC, however, has key advantages: it provides a times-series (1990, 2000, 2006) and was designed for temporal and spatial consistency. Time series of consistent, more detailed national land use maps are scarce, the presented Dutch case being an exception. In effect, detailed national land use datasets are often not comparable (between years and between countries) due to different nomenclatures and mapping protocols. On the other hand, the drivers of the development of industrial, commercial and services land uses are difficult to grasp. Does economic development lead to spatial impacts or the other way around? Or, is there a more complex non-linear interaction between land use change and regional economic performance? These questions, although legitimate and pertinent, were not the focus of this study.

Given the high correlation between the economic output of a region and its total industrial, commercial and services land use, we started out assuming a direct and linear relationship between those two variables. Four methods based on land use intensity measures were devised and compared to simple trend extrapolation techniques in a case study for South and Western European countries. All models were, in addition, compared with the null model, which assumes no land use change during the validation time interval. The models were calibrated using information for the period 1990–2000 and then used to estimate the observed industrial and commercial land use in 2006, as reported in the CLC.

All models performed substantially better than the null model, which indicates that any of the devised modelling approaches is better than not modelling at all. However, none of the land use intensity approaches consistently outperformed the linear trend. Results seem to indicate that simpler assumptions to estimate industrial and commercial land return overall higher accuracies at least for short-term estimations. Nonetheless, by analysing the validation results at the country level we cannot discard that approaches based on land use intensity measures yield superior accuracies for many regions and even whole countries (see table 6). It seems, indeed, that there is not a one best approach for the entire set of tested countries.

The linear extrapolation has slightly produced less error dispersion when compared to other methods, despite the fact that it does not integrate any actual independent driver of land use change. As the estimation period extends, the high performances of the linear extrapolation model may phase out quicker than those of any model which relies on the economic activity as a proxy for land use changes. While for short-term estimations the linear extrapolation might actually produce very plausible overall estimations, for the medium and long term estimations, simple linear trends are thus conceptually unacceptable.

It is worth exploring the reasons why approaches based on intensity measures did not work as well as one could have expected at the start of this study. Even though GVA and total industrial and commercial land use are highly correlated at NUTS2 level, it cannot be inferred that change in GVA and change in land use are equally correlated. Changes in the economic structure will certainly lead to changes in the ratio between economic output and required land. On top of that we can expect time-lags between economic developments and their spatial impact and vice-versa. Old industrial sites (brownfields) may wait decades for redevelopment and thus remain present in the landscape long after their economic activities cease. While on the other hand expected economic development may lead to the construction of new offices and business estates that may remain empty for years. As mentioned earlier, complex issues like these call for more in depth study of the spatial development of industrial and commercial land use and its interaction with the underpinning economic drivers. Finally, the methods based on regional and sector specific land use intensity did not perform better than those based only on regional specific land use intensity. The failure to obtain better results for these particular methods may be at least partially explained by the weaknesses in the correspondences between a) the NACE classification, in which the GVA data is based, b) the CLC nomenclature, and c) the nomenclature of the two national land use datasets. All these three elements were required, first to disaggregate the sectorial composition of CLC class ‘industrial and commercial units’, and second, to estimate regional and sector-specific land use intensities. In fact, the assumptions made when coupling different nomenclatures may have led to uncertainties and errors that propagated to the final results of models 5 and 6.

The use of static land use intensities, as measured in the calibration years, was yet another drawback. In fact, the assumption of stable land use intensities has contributed to the overall error of these approaches. For the model 5 in particular, a factor *ω* was introduced, allowing for change in the land use intensities. However, in this case study, the factor was set to 1 in order to have a neutral effect on the land use intensities. Later, by making *ω_s_* a function of the observed changes in the sectorial land use intensities between 1990 and 2000, we observed a substantial increase in the accuracy of the model 5. The total relative difference decreased to −4.78%, the average absolute error decreased to 572 ha, and the total absolute error dropped to 6.51%, thus making M3 and M5 very close in terms of overall accuracy. This demonstrates how important it is to account for temporal changes in the land use intensities, rather than keeping them static. However, the study of the changes in land use intensities, their trends and drivers, is yet to be made. Unfortunately, the available data at European level is yet insufficient for an appropriate assessment. Finally, even if more detailed land use maps are available for some European countries, consistent time-series are still lacking.

Another intriguing aspect stood out from the validation results. All models have underestimated the amount of industrial and commercial land use in 2006 (table 5). We tested the hypothesis that the observed consistent underestimation was at least partially a consequence of issues related to the input land use data from CLC. By applying all six methods to the Dutch regions using a more detailed national land use source, substantial reduction of deviations was observed for all models, and the resulting relative differences between known and estimated land use were much less biased, with models 1, 2 and 3 actually producing slight overestimations (see table 7). These results, although referring to a small portion of the entire case study, demonstrate how sensitive the methods are to the detail and accuracy of the input land use data.

Finally, to illustrate the practical application of the land use intensity-based methods, we used model 3 (best performer among all the tested models) to project industrial and commercial land use demand, given a hypothetical scenario of linear economic growth up to 2020 for the Portuguese NUTS2 regions. While implementing this ‘forecasting’ exercise, we identified the main sources of uncertainty related to the model used. The land use intensity parameter itself, which was measured for the year 2006 using the CORINE Land Cover and economic statistics from Eurostat, was found to be uncertain due to inaccuracies in the land use mapping. Based on a statistically sound validation of the CLC 2006 for Portugal, it was possible to draw a 90% confidence interval around the land use intensity for each region. In addition, we proposed two extreme scenarios for the evolution of the regional land use intensities, based on past trends. From one single hypothetical economic scenario, we arrived to 5 possible trajectories of future industrial and commercial land use demand, confirming that the uncertainties can be substantial.

Despite the limitations herein summarized, we argue that straightforward approaches, such as the ones based on land use intensity measures were lacking, and are relevant and suitable for large study areas, where data are limited. Whereas the uncertainties of these methods could be narrowed in part by using more detailed and consistent land use time series data, the uncertainties related to economic forecasts will remain and are intrinsic to the source of the forecast (i.e. economic models). The likely future evolution of regional land use intensities remains unknown for the most part, and more detailed studies are needed to grasp the underpinning factors. However, the dynamic change in land use intensities could be addressed, for instance, as proposed in equation 11, or through any other suitable variant. The *ω* factor can be the result of a calibration process or used as a ‘policy parameter’ in the context of scenario analyses and ex-ante impact assessment of policies. In sum, the proposed methods allow the generation of sensible and scenario-dependent results, on developments of industrial and commercial land uses across regions, which are linked to macro-economic models. As for the limitations and uncertainties, they should be acknowledged and dealt with transparency, as in any other modelling exercise.
